# A ROS2-Based Gateway for Modular Hardware Usage in Heterogeneous Environments

**DOI:** 10.3390/s24196341

**Published:** 2024-09-30

**Authors:** Rúben Carreira, Nuno Costa, João Ramos, Luís Frazão, António Pereira

**Affiliations:** 1 Computer Science and Communications Research Centre, School of Technology and Management, Polytechnic of Leiria, 2411-901 Leiria, Portugal; 2220661@my.ipleiria.pt (R.C.); nuno.costa@ipleiria.pt (N.C.); joao.f.ramos@ipleiria.pt (J.R.); luis.frazao@ipleiria.pt (L.F.); 2 INOV INESC Inovação, Institute of New Technologies, Leiria Office, 2411-901 Leiria, Portugal

**Keywords:** ROS2, robotics, IoRT, interoperability, modularity, payloads, technology, communication, integration, middleware

## Abstract

The rise of robotics and the Internet of Things (IoT) could potentially represent a significant shift towards a more integrated and automated future, where the physical and digital domains may merge. However, the integration of these technologies presents certain challenges, including compatibility issues with existing systems and the need for greater interoperability between different devices. It would seem that the rigidity of traditional robotic designs may inadvertently make these difficulties worse, which in turn highlights the potential benefits of modular solutions. Furthermore, the mastery of new technologies may introduce additional complexity due to the varying approaches taken by robot manufacturers. In order to address these issues, this research proposes a Robot Operating System (ROS2)-based middleware, called the “ROS2-based gateway”, which aims to simplify the integration of robots in different environments. By focusing on the payload layer and enabling external communication, this middleware has the potential to enhance modularity and interoperability, thus accelerating the integration process. It offers users the option of selecting payloads and communication methods via a shell interface, which the middleware then configures, ensuring adaptability. The solution proposed in this article, based on the gateway concept, offers users and programmers the flexibility to specify which payloads they want to activate depending on the task at hand and the high-level protocols they wish to use to interact with the activated payloads. This approach allows for the optimisation of hardware resources (only the necessary payloads are activated), as well as enabling the programmer/user to utilise high-level communication protocols (such as RESTful, Kafka, etc.) to interact with the activated payloads, rather than low-level programming.

## 1. Introduction

The advent of robotics and the Internet of Things (IoT) has brought about a new era of connectivity and automation, which has had a notable impact on the way we interact with the technology around us. This era is distinguished by the convergence of the physical and digital realms, where robots and IoT devices collaborate to automate tasks, enhance efficiency and improve the overall quality of life. In the view of D. Villa et al. in reference [1], the concept of the Internet of Robotics Things (IoRT) has emerged, which has the potential to enable robots to be integrated into IoT ecosystems, facilitating communication and automation across a wide range of applications. In these ecosystems, each device is supposed to be treated as a thing, meaning they must enforce interoperability and communication standards, allowing for seamless integration of robots with other robots, sensors, servers and so on. This can often present a number of challenges. One of the key challenges is the learning curve and the need for adaptation to the device’s technology. In some cases, adapting to new technologies and methodologies in environments where legacy systems are in place [2] can be particularly challenging. It often requires significant time and resources, which may lead to implementation delays, increased costs and disruptions to existing workflows. Furthermore, this can be particularly challenging in environments with a variety of different devices, as it can be difficult to integrate and coordinate robots and other devices from different manufacturers [3].

Another challenge is the limitation of robots to the same hardware and features, which may potentially restrict their adaptability and flexibility in performing diverse tasks and services. It would seem that there is an increasing demand for robots that are versatile; therefore, it is becoming increasingly important for robots to be able to adapt their hardware payloads in a modular way [4]. Monolithic robots may not be as adaptable as we would like them to be, particularly in different environments and tasks. Additionally, robots are usually built to have all their payloads always be active, regardless of the task at hand. This can sometimes result in inefficiencies and lead to faulty hardware, which may in turn lead to increased costs and delays. It would be beneficial to consider ways to enhance the flexibility of robots in order to avoid the need for configuration and integration of new robots into the current system. This would help to address the previous challenge and improve the overall efficiency and cost-effectiveness of the process. The concept of modular robots offers a potential solution to this issue by allowing robots to activate or deactivate their hardware components based on the task requirements, as if the payloads or modules were on-demand plug-and-play. It would seem that, according to reference [5], there have been efforts to create standards in robot modularity. A recent ISO standard [6] has been published, providing guidelines for the world of modularity in robotics or IoRT systems. Despite the considerable scientific research that has been conducted in this area, it would appear that few concrete breakthroughs have been accomplished.

This article explores the potential of a Robot Operating System 2 (ROS2)-based middleware to address the challenges previously mentioned. ROS2 is a set of widely used software libraries and tools for building robot applications. The implemented solution leverages a set of ROS2 packages operating at the robot’s payload layer and provides bridges for the external communication layer where the robots communicate with external sources/clients on-demand. It is worth noting that traditional robots are often set to activate or power on all the payloads currently connected to the robot, regardless of whether they are necessary for the task at hand. This can result in a waste of resources. The main contribution of this article is the possibility for the programmer or user to choose (in a user-friendly way) which payloads should be active depending on the task to be performed. This can help to optimise the use of resources. Additionally, the possibility of choosing the high-level communication protocol for interacting with the robot and active payloads (RESTful, Websockets, Kafka, etc.) can further enhance the efficiency of the system. This could free the programmer from low-level programming. Therefore, it is our intention to make the robot’s workflow as modular and agnostic as possible for the user, while also greatly increasing its interoperability levels. Through a shell user interface (UI), users have the option of inserting their physical environment’s settings and needs, which the middleware will then adapt to, thus enabling a faster and more agile robot integration process. In particular, users have the option of selecting a number of payloads and communication technologies from the list of those available for the robot, depending on the tasks they wish to perform and the systems they intend to integrate with. Once the selection process is complete, users can then define the necessary configurations for each chosen payload and communication technology. The system will then launch the required ROS2 packages (behind the scenes) to match the users’ needs and configurations and provide the required usage documentation.

By focusing on modularity (loading payloads on-demand) and interoperability (through high level communication protocols) at the payload level, this work offers a novel approach to configuring and deploying robots in various environments and tasks. This modular design has the potential to facilitate easier adaptation to changing requirements and may also help to significantly reduce the overhead associated with integrating new technologies or adapting to legacy systems. Furthermore, the introduction of a shell UI for configuring robot payloads and communication technologies represents a notable step forward in terms of user-friendliness and agility in robot deployment, eliminating the need for low-level programming. This feature offers users the opportunity to adapt their robot setups to suit their specific requirements, which could potentially enhance the overall effectiveness and efficiency of ROS2-based projects.

The article is structured as follows: In Section 1, we endeavour to describe the challenges that we believe to be the most pertinent, and to provide an overview of the solution that we have developed. Section 2 is intended to provide an overview of the technologies employed during the development of this solution, as well as to address the research work and relevant related systems. Section 3 attempts to provide an overview of the architectural approach, logic and terminology used in this solution. We also endeavour to provide a more detailed description of the developed system. The following section, Section 4, considers the potential applications of the proposed solution in a variety of contexts. Finally, Section 5 considers future developments and improvements, and offers some concluding remarks.

## 2. State of the Art

### 2.1. Background

The majority of this solution’s components were developed using the ROS2 framework, as it is a widely known and well-established platform that offers a large set of controllers, algorithms, libraries and useful features such as APIs for parameters [7]. It goes without saying that ROS2 forms the bedrock of the entire solution. It would be beneficial to provide an overview of the concepts underlying this framework, particularly in the context of the development phase.

#### 2.1.1. ROS2 Architecture

ROS2 has the potential to enhance the Data Distribution Service (DDS) middleware by introducing a DDS abstraction layer, which could simplify the interaction with the DDS APIs for users, as outlined in [8]. This abstraction layer could streamline high-level configurations and optimise the utilisation of DDS. Furthermore, the adoption of DDS in ROS2 could obviate the necessity for a master process, which could be a significant feature that bolsters fault tolerance. These features were important considerations when choosing this technology.

#### 2.1.2. ROS2 Package

In the context of ROS2, a package can be understood as an organisational code unit. They have been designed with the intention of making the installation, sharing and building of ROS2 projects as straightforward as possible. One might consider it to be a container for all the components of a project, including the source code, libraries and executables. A package makes it much easier for developers to work together, allowing them to use and build projects from other developers and then integrate them into other projects. They are designed to follow a specific structure and use build tools such as colcon, which offers support for CMAKE and Python. ROS2 provides a command line tool with a variety of commands for building and using packages. This structure and organisation facilitates more effective management and distribution of ROS2 projects for developers.

#### 2.1.3. ROS2 Topics

In ROS2, a topic can be seen as a conduit for publish/subscribe interactions, which allows data disseminated by one or more nodes to be accessible to other nodes that have subscribed to the specific topic. The data exchanged through a topic can be organised into a set of attributes, which the nodes aiming to communicate over the topic should be familiar with.

#### 2.1.4. ROS2 Launch File

An ROS2 launch file is a useful tool that can help to streamline the process of starting, configuring and managing multiple ROS2 nodes simultaneously. It is used to describe the configuration of ROS2 systems, including which packages or programs to run, where to run them, what arguments they require and so on. These launch files can be written in Python, YAML or XML, providing flexibility in how developers want to define and manage their ROS2 applications.

As highlighted in reference [9], ROS2 offers a set of well-defined principles, aligns with modern requirements and provides extensive support worldwide. These attributes position it as a leading choice in the robotics field. We believe that this solution, which employs ROS2 features, will contribute to the growth of the framework’s community.

### 2.2. Related Work

According to references [10,11,12,13], when it comes to the IoRT field, the current research regarding hardware modularity and adaptability is mainly focused on how robots can change their physical form to adapt to given tasks while optimising their energy and resource consumption. It would appear that there is currently no public research related to on-demand hardware usage and communication protocol interoperability with the aid of ROS/ROS2. This may indicate that this particular aspect of robotics is not yet receiving the attention it deserves.

This project proposes the use of the ROS2 launch file feature to facilitate the “bring up” concept, which is beneficial for launching the required packages. It would be remiss of us not to mention some other research works that employ this concept.

The article “Exploring a Supervisory Control System Using ROS2 and IoT Sensors” [14] offers insights into the development of a supervisory control system for additive manufacturing (AM) systems, with a particular focus on the integration of ROS2 and IoT sensors for enhanced process monitoring and control. The system is designed to help address the challenges posed by the variability and unpredictability of AM processes, particularly in wire-fed directed energy deposition (DED)—a method that requires precise control and monitoring to ensure quality outcomes. The research highlights the value of process monitoring in AM given the potential for defects to arise from inconsistencies in metal properties and deposition processes. It suggests the use of computational nodes linked to various sensors, including robot controllers and thermal monitors, as a potential means of gaining deeper insights into system health and process parameters. It would be fair to say that these nodes are managed by the ROS2 architecture, which facilitates their connectivity and operation.

The article [15] introduces a novel embedded robot system designed for cloud robotics, which appears to be an innovative approach leveraging “ROS2 on Yocto” to support various robotic applications. The heart of this system is a lightweight operating system built on the Yocto Project, which has been selected for its scalability and portability across different hardware and software configurations. This approach has the potential to facilitate the development of a robust platform suitable for low-specification embedded systems, which are typical in robotics. The system incorporates a number of advanced features, including Simultaneous Localization and Mapping (SLAM), navigation, path planning and motion control. These have been validated through a number of real-world applications, including home cleaning and indoor delivery robots. It is evident that these robots are capable of autonomous driving, obstacle detection and avoidance, which serves to demonstrate the effectiveness of the proposed system.

The software architecture of the thin client robot is structured across five layers, which we believe to be the optimal number for the system. We would like to suggest the following additional layers: Yocto Project, meta-3rd party, metal-geros2, meta-chipset and meta-lg. Each layer has a specific purpose, from providing open-source ROS2 packages to integrating chipset-specific drivers and optimising the system for embedded environments. This modular design allows for the swift updating of software versions and lends itself to a variety of form factors, combining common components with those tailored to specific chipsets.

In reference [16], HyperDog is introduced, which is an open-source, quadruped robot platform that has been designed with the intention of facilitating research and development in the field of legged robotics. Its construction primarily makes use of 3D-printed parts and carbon fibre, resulting in a lightweight yet sturdy robot. The platform is built upon ROS2 and micro-ROS, providing a convenient framework for developing and testing various locomotion algorithms in both simulated and real-world environments. The robot’s control system makes use of micro-ROS for low-level operations, which allows for seamless integration with ROS2-based systems and enhances the robot’s responsiveness and reliability. The paper touches on some of the challenges that have been encountered in the development of quadruped robots, particularly the need for efficient locomotion algorithms and the limitations of existing open-source platforms. It is the authors’ hope that by presenting HyperDog, they can help address these challenges and offer a versatile platform for researchers and engineers to explore the full potential of legged robotics in various applications, from search and rescue to industrial inspection.

In reference [17], FogROS2 is described. It builds on the original FogROS1 framework with the aim of fostering closer collaboration between robotics and cloud computing. FogROS2 has been developed with the aim of facilitating cloud and fog robotics within the Robot Operating System 2 (ROS2) ecosystem, with the intention of addressing the challenge of executing computationally intensive robot algorithms efficiently. FogROS2 has the potential to offer significant performance gains in common robot applications, which could, for example, lead to reduced SLAM latency. This new development helps to overcome the challenge that robots face in keeping pace with advancements in algorithms and computing hardware. It offers on-demand access to extensive computing resources, including GPUs, TPUs and FPGAs, through cloud computing. The introduction of FogROS2 represents a notable evolution from FogROS1. It has been developed from the ground up to seamlessly integrate with ROS2, leveraging its enhancements in networking, launch configurability and command-line interface. Additionally, FogROS2 has the potential to integrate with Foxglove for remote monitoring, which could simplify the deployment of robot code in the cloud and extend its applicability to a wider range of robot applications. By leveraging cloud computing, FogROS2 demonstrates how latency in complex computations can be significantly reduced. However, it is worth noting that certain computations may not be suitable for the cloud due to unpredictable network times.

Given that ROS2 is open-source, meticulously maintained and utilised globally, it has attained a commendable level of stability in its features and documentation, which makes it a highly regarded option in comparison to other alternatives. ROS2 has the advantage of offering a complete ecosystem specifically designed for robotics, which is easy to use, flexible and scalable. ROS2 is unique in that it is built around a decentralised architecture, which allows it to support a wide range of hardware and software. This facilitates seamless interoperability and integration and enables a modular design, which allows users to break systems down into smaller and reusable components. This provides good performance in large-scale projects. It would be remiss of us not to mention another key aspect of ROS2, namely, its communication method. It introduces advanced communication mechanisms, including reliable and unreliable messaging, which could be seen as crucial for handling dynamic robotics environments. While the aforementioned projects are not directly related to the one presented in this article, they do represent notable advancements in several other fields with the aid of ROS2. We believe that ROS2 is a good choice when considering the development of a robotic solution. It is also worth noting that ROS2 launch files are a popular choice, as they offer a strategic way to easily turn on entire systems with one command while also enabling interoperability and scalability. It is possible that launch files may group several ROS2 packages, which could be beneficial in certain scenarios. It might be said that these projects make use of the “bring up” concept in order to enable communication among packages inside the ROS2 domain. They primarily enable the activation of ROS2 libraries or software modules for the purpose of controlling hardware. It might be suggested that the ROS2-based gateway introduces a new purpose for the “bring up”. It has the additional benefit of enabling cross-domain interoperability, as it allows ROS2 messages to be bridged to several other external communication protocols and vice versa.

In addition to the strengths of ROS2 that have been highlighted above, we believe that the gateway developed for this project further enhances the capabilities of ROS2-based solutions by addressing specific challenges in the integration and operation of robots within any ecosystem. We hope that the work presented here will complement the ROS2 projects listed above by offering practical solutions to common challenges faced in robotics and IoT integration. By making use of the strengths of ROS2, this project aims to contribute to the advancement of robotics development, setting a benchmark for future innovations in the field. This gateway development addresses a crucial but previously overlooked aspect of the IoRT field, namely, the underexplored area of on-demand hardware usage and communication protocol interoperability. While the broader research community has made significant strides in enhancing robot hardware modularity, as well as integrating ROS2 with various IoT sensors and systems, there appears to be a notable absence, to the best of our knowledge, of targeted solutions for dynamically adjusting robot payloads and communication technologies based on task requirements. We believe that this project can help to address this gap by introducing a middleware that utilises the ROS2 launch file concept to enable a more agile and flexible deployment of robot functionalities. In this way, we hope that our work will complement and expand upon the achievements of other ROS2-centric initiatives.

## 3. Architecture and Prototype

**Figure 1:**
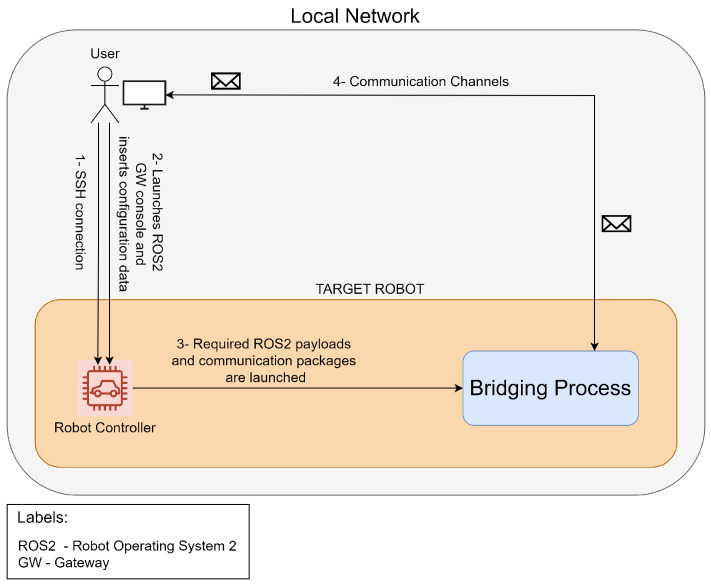
Solution’s workflow.

As previously mentioned, this solution aims to ensure a fast and agnostic integration of robots into any kind of technological environment, while enabling the modular usage of the robots’ payloads on-demand. It should be noted that there are numerous communication protocols in use, with new ones emerging frequently. In light of this, the approach taken with this solution was to enable interoperability with the most commonly used technologies, with the flexibility to add further ones in the future. We hope that Figure 1 will help the reader to understand the general workflow of the developed solution. The process begins with the user accessing the robot’s platform via a Secure Shell (SSH) connection and initiating the gateway’s launcher script. Once the configuration has been provisioned, the user is free to proceed without further action. The gateway is responsible for launching the required ROS2 packages regarding the required payloads and communication technologies, and for ensuring everything is configured according to the data provided by the user. Once this process is complete, the user will be provided with all the required endpoints and connection information, allowing he/she to start using the robot for whatever purpose they require. It might be helpful to clarify that once the robot is up and running, it is not configured to perform any kind of task. The objective here is to enable external access and control of each payload so that users may program them according to their needs in a modular and high-level way using the technologies they are already proficient in.

### 3.1. Logical Architecture

**Figure 2:**
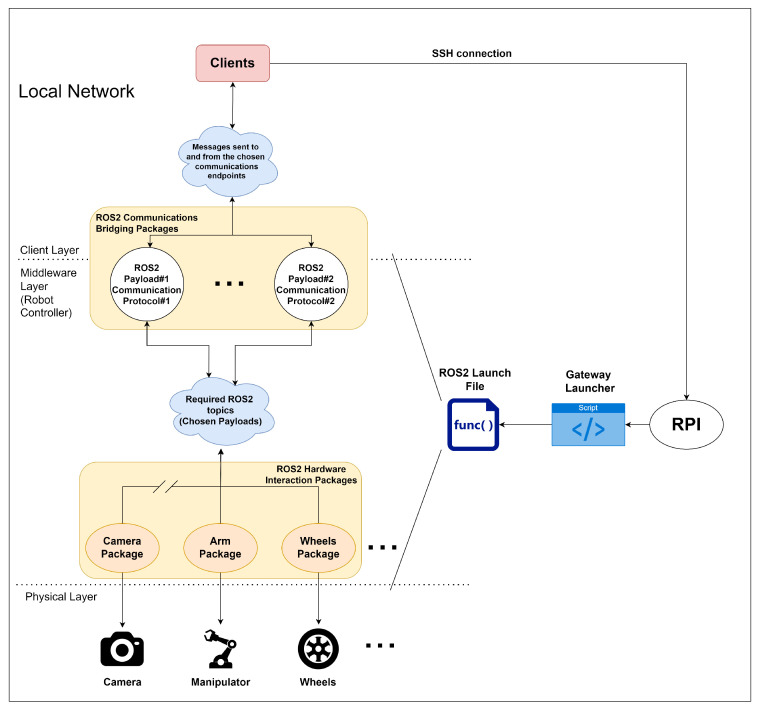
Gateway’s architecture.

If we might suggest, this solution could be divided into three main layers, which might make it easier to identify the relationship between each entity in the architecture. For a more detailed understanding of these layers, we would like to kindly direct you to Figure 2.

- **Client Layer**—this layer is where the clients are represented. It should be noted that entities in this layer are limited to the external control of the target robot. Therefore, it is understood that the underlying processes and remaining features fall outside of their scope. Consequently, there are endpoints and topics that are visible to the client side, which also form part of this layer.
- **Middleware Layer**—this is the main layer of the architecture, which serves to house the core components of the solution. From the initial launcher script, which defines how the robot will behave, to the actual ROS2 packages orchestration, taking into account the user input, everything is handled in this layer. The aim is for all of these processes to be transparent for the users.
- **Physical Layer**—this layer represents the physical attachable components, which are then used as the robot’s payloads through ROS2 packages. It would be fair to say that these packages play a role in converting ROS2 messages to the applicable payload low-level communication protocol.

### 3.2. Non-ROS2 Important Concepts

In addition to the ROS2 components used in this solution, there are also some fundamental concepts outside the ROS2 environment that could be considered as potential channels for indirect interaction with it.

#### 3.2.1. Gateway Launcher Script

A bash script that serves as the entry point to the entire mechanism. It serves as a user interface, enabling users to interact with the solution in a more intuitive and accessible manner. Once the script has been launched, users are invited to go through a series of steps in order to provide context to the gateway. This ranges from the payload or payloads that need to be activated to the high-level communication protocols that need to be followed in order to interact with the target robot. Subsequently, the ROS2 launch file is employed to initiate the process, with the user-provided arguments and parameters duly considered.

#### 3.2.2. Bridging Process

It is this concept that makes this solution a valuable asset in robotic systems. Its purpose is to facilitate the conversion of messages from a given high-level communication protocol to ROS2. In other words, it acts as a conduit, enabling the solution to comprehend and interact with a structure that may otherwise be perceived as foreign. This ultimately paves the way for seamless interoperability between the robot and its surrounding environment. It might be helpful to note that each payload has several ROS2 packages assigned, with each package representing a different bridge. If we might suggest, a robot with a robotic arm payload would also require a hardware communication package to enable the arm to function. In addition, there are packages for communication through several widely used technologies, such as websockets, REST, Kafka, CoAP, MQTT and so on. Once the gateway mechanism has been launched in accordance with the user’s configuration, these packages assume responsibility for enabling the arm payload to be controlled through one or more of these technologies. This is because, upon receipt of a message at the gateway, it is then translated and sent to the appropriate ROS2 messaging topics. It is also important to be aware that payloads may communicate through completely different protocols. As an example, cameras may require streaming technologies, so it would be advisable for each payload to have its own adapted ROS2 communication protocols.

### 3.3. Implementation

This section aims to provide some insight into the implementation phase. In this section, we will explore the entire development stage, up until the first stable prototype is created. We will look at the process from the initial architecture definition through to the last modifications and the thought process behind each step.

#### 3.3.1. Prototype’s Architecture

**Figure 3:**
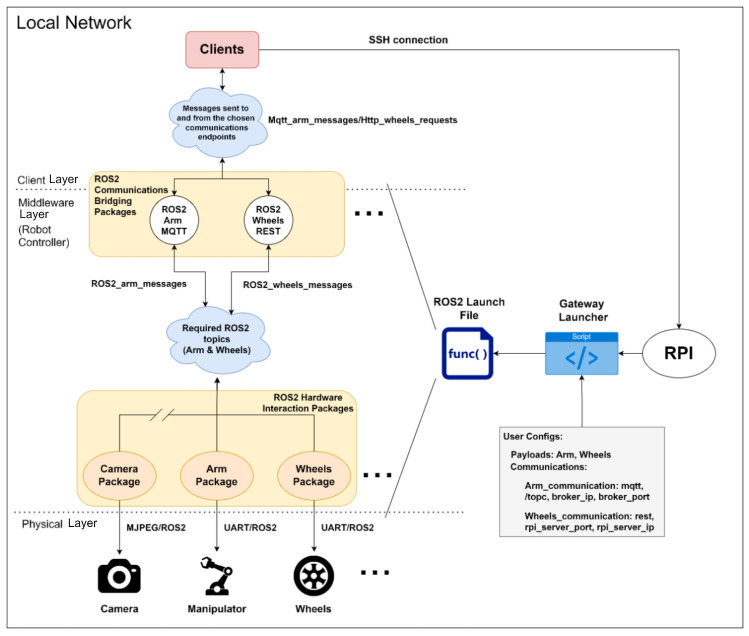
ROS2-based gateway architecture exemplifying a use case.

Once the issues had been identified and solutions found, it was time to consider how the gateway could be brought to life through the design of a well-structured architecture. After considering a number of possibilities, it became clear that the architecture should be designed to accommodate a wide range of use cases and be sufficiently straightforward to be easily replicated. As a result, the components were divided into the logical layers that had been previously mentioned, which helped to simplify the mental representation of each stage of the workflow. Please refer to Figure 3 for a representation of the whole architecture as a use case, which illustrates how the gateway behaves from the bottom to the top when users/programmers decide to bring up certain payloads. In this particular instance, the diagram illustrates how the components work when the user elects to utilise both the arm and wheels payloads concurrently.

#### 3.3.2. ROS2 Launch File Development

It could be considered the core of the whole mechanism. This Python file has the potential to orchestrate the entire ROS2 domain. It would appear that the input parameters and arguments are taken from the user via the Gateway Launcher Script UI; then, some logic is used to add the ROS2 packages to an array. When adding the packages to the array, this file also fills the code placeholders during runtime with the data the user requires in terms of connection settings (IPs, ports, topic names, URLs, etc.). Once this process is complete, the launch file returns the package array, which is then used by ROS2 to launch and make each package available. To execute this file, one just has to build its own package using the colcon tool and then use the “ros2 launch” command [18].

As an example, when a user executes the initial user interface and chooses what payloads and communication technologies he/she intends to use, the UI shell script runs the ROS2 launch command to process the launch file. The launch file’s logic is prepared to take into account two arrays: one array containing the target payloads and another array containing the intended communication technologies. The program then checks, for each inserted payload name, whether there is an available ROS2 payload package mapped to that name. If there is a corresponding payload package, the program then loops through the inserted communication technologies’ names array and attempts to ascertain whether there is an ROS2 communication package available for that payload that is mapped to that given communication technology name. If a match is found, the program adds the respective package and its parameters (inserted by the user and mapped to code placeholders in the parameters property of the package) to the node’s array. Once the arrays have been looped, the program executes the resulting nodes array.

**Figure 4:**
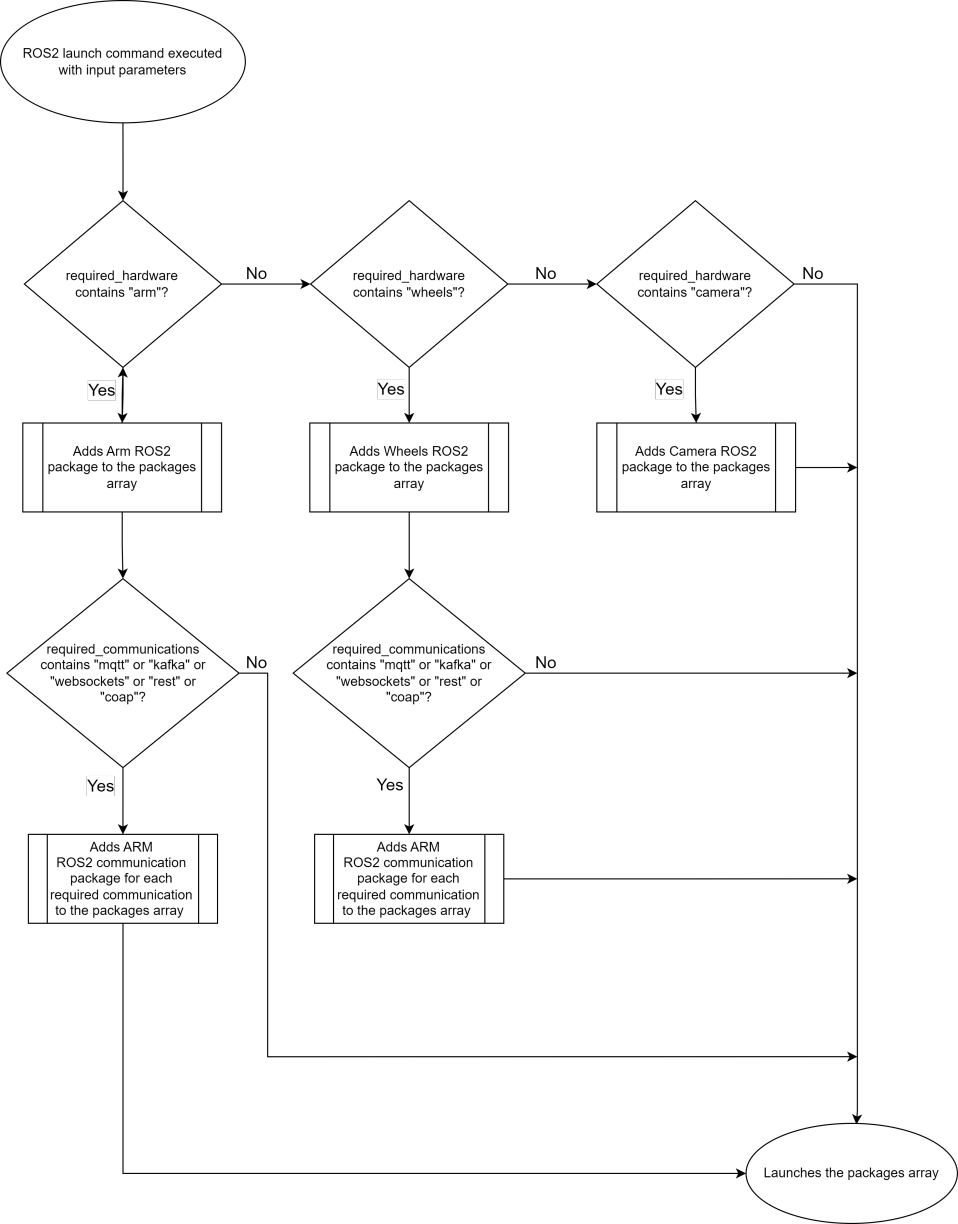
ROS2 Launch file flow diagram.

By referring to Figure 4, it may be possible to gain a deeper insight into the logic behind the launch file. On closer inspection, it becomes apparent that the logic governing the camera payload is somewhat distinct, largely due to the constraints imposed by the hardware. Given that it is a camera, it communicates through different methods, so a different approach was required. We would like to draw your attention to the GitHub repository in reference [19] that has been created with the aim of developing an ROS2 package that can accept ROS2 image frame messages and convert them into a variety of streaming codecs, which can then be made available through a web server. The streaming protocol can be selected through the web server’s Uniform Resource Locator (URL). It is then possible for the client to manipulate the web server’s URL in order to suit their requirements. It may be the case that different approaches are required for other payloads as well. There is no standard solution, so users are free to adapt the gateway to their needs. If the users require additional payloads for any reason, they are kindly requested to add the respective hardware and communication packages to this logic so that the launch file can take them into account.

#### 3.3.3. ROS2 Payload Packages

As previously mentioned, these packages contain the code and related dependencies. ROS2 offers a modular and distributed framework, which provides the potential for an ROS2-based system to be built upon several ROS2 packages that can communicate with each other. Hence, the solution provided in this article is not fundamentally different. It draws upon ROS2 packages to facilitate the integration of hardware into the ROS2 domain, thereby ensuring accessibility. The ROS2 hardware packages are constructed in a way that allows for the physical connection from the payload to the Raspberry Pi (or any other type of computer) to be made as intended. As an example, if a payload is communicating through universal asynchronous receiver/transmitter (UART), it would be advisable for the ROS2 package to be developed in a way that allows messages from the lower level to be converted to standardised ROS2 messages and vice versa. Given this conversion, it would be beneficial to create ROS2 nodes with the necessary publisher and subscriber objects to facilitate communication through message topics. Once this process is complete, it may be advisable to test the package running it as standalone using the “ros2 run” command.

These ROS2 payload packages are stored locally on the robot, specifically on the created ROS2 workspace. While the architecture could be designed in a distributed way, for example, by introducing cloud concepts to reduce loads, it was thought that this solution should be as independent as possible right from the beginning. Therefore, it was decided that the packages would be stored locally to prevent several points of failure.

#### 3.3.4. ROS2 Communication Packages and Bridging Process

Communication packages are what allow the robot to be integrated in several environments. Communication packages are just like the payload packages regarding its constitution but the logic is different. These packages introduce some translation processes at a higher level and then pass the data downstream to the lower levels until they reach the physical layer. As an example of data flowing downwards, first, these packages open a communication channel to the outside (as long as it is inside the same network). Then, depending on how the logic is applied, the idea is to handle messages coming from the clients, convert them into an ROS2 messages and send them to the respective ROS2 payload’s topics. In a use case where the wheels payload has an ROS2 hardware package with a subscriber object subscribed to a topic called “/control_movement”. If the wheels’ websockets package is running, it connects to a given websockets server and upon new messages from that server, the logic performs the bridging process by handling the received data and publishing it to the “/control_movement” topic so the hardware package may control the wheels accordingly.

These packages are also stored locally as stated above in the ROS2 Payload Package subsection.

#### 3.3.5. Bash User Interface

With the aim of providing a more user friendly experience, a bash script was created.

It is meant to provide some abstraction levels to the ROS2 environment, guidelines and a more structured way of visualising information during the provisioning phase and after launching the gateway, making it simpler to use this solution, especially when the end users are less experienced in the field.

This is a straightforward user interface component that aims to facilitate the use of the gateway by users. Its primary objective is to request information from the user, such as the necessary payloads and communication protocols. For each selected communication protocol, it then requests connection settings. Subsequently, the “ros2 launch” command is executed in order to call the launch file, with the user data passed as parameters. Finally, once everything is operational, it provides all the necessary information for the user to understand how to access and use the gateway, taking into account the selected options and the provided connection details.

The idea is that the user/programmer may find it more convenient to interact with the robot without having to use low-level programming and to avoid wasting resources by always having all the existing payloads switched on even if they are not needed. This is because the user now has a console that defines which payloads should be active and a high-level communication protocol that the user can choose, based on their references, to interact with the robot/payloads. This should result in savings in resource consumption and provide a uniform means of interacting with the target robot/payloads.

In conclusion, this mechanism offers users the flexibility to adapt it to their specific needs. Since each payload will have its specific requirements, it is hard to come up with a standardised way of adding and configuring newer ones; so, it is up to the user’s creativity to decide how it is intended to be coupled with the gateway, making this solution flexible enough to adapt to specific environment constraints. Provided that each payload is coupled with an ROS2 payload package that provides a way to control it and an ROS2 communication package matching the user’s current communication technology, the gateway will be able to integrate the new payload and make it available to external sources/destinations. Additionally, we recognise that the initial version of this solution may require some time to manage and configure multiple payloads and high-level communication protocols through the UI component. However, this is a one-time process for a given task, and its aim is to provide a more user-friendly, high-level way for end-users to provision the target robot without the need for low-level programming. Nevertheless, it is our intention to modify this human–robot interaction in order to ensure that payloads and communication protocols can be provisioned more quickly in future iterations.

## 4. Results

**Figure 5:**
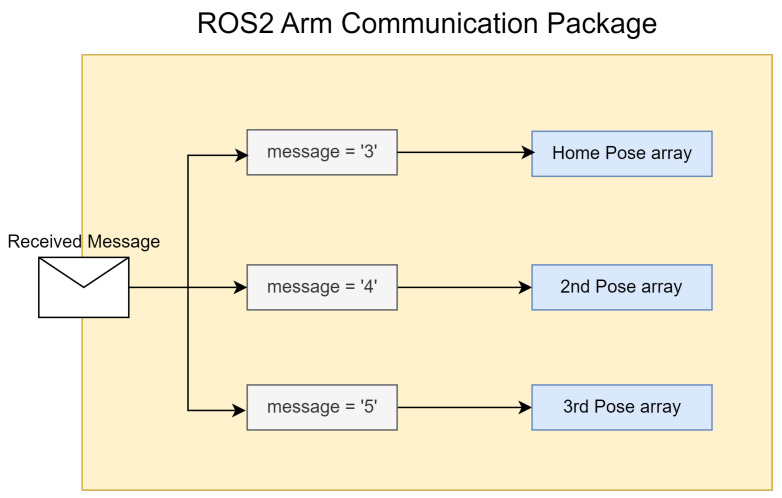
ROS2 Arm received messages logic.

This section aims to provide a comprehensive overview of the most relevant tests conducted during and after the implementation phase. The structure is designed to reflect a logical progression of test complexity, beginning with the most straightforward tests and gradually progressing to near-real case scenarios. For the purposes of testing and proof of concept, the messages received by the arm payload have been simplified as much as possible. As an alternative approach, we have opted to simplify the replication of ROS2 topics into endpoints and external topics, and to communicate through increments/decrements on the arrays of coordinates or joint angles that the arm uses to move. Instead, we have defined three main arm poses, as illustrated in Figure 5. It might be helpful to note that each pose has its respective static joint angle array. Upon receiving messages from external sources, if the message content corresponds to one of the three values defined to map to each pose array, then this array is sent to the arm control topics.

It is worth mentioning that when the development of the ROS2-based gateway started, the most stable and used ROS2 distribution was ROS2 Foxy Fitzroy. Since the prototype was developed and tested on the TurtleBot3 available at the time, and its documentation used ROS2 Foxy as the basis, this is the distribution that is currently used. Since the release of ROS2 Humble, the TurtleBot3 documentation has undergone some updates; however, unfortunately, the specific robotic arm purchased to integrate with TurtleBot3 still works with ROS2 Foxy, so it was decided not to update the ROS2 version to avoid unpredictable behaviour.

### 4.1. The Select and Interact with Payloads Test Case

**Purpose:** The The aim of this test was to ascertain whether the gateway is functioning as intended, enabling the selection and interaction with a chosen payload. This should demonstrate that the mechanism allows for payload modularity and adapts the robot to an on-demand communication technology.
**Test bed:** Table 1 presents a list of resources required to perform this test.
**Results:** The test case has shown that the gateway is able to provide on-demand payload usage and a high-level communication protocol as well.

**Figure 6:**
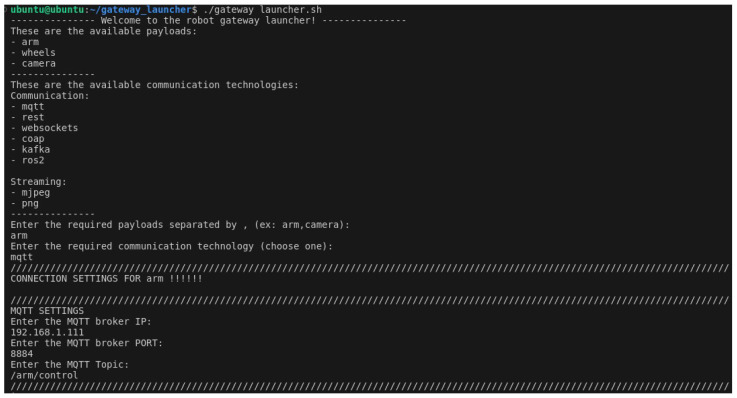
User input for the select and interact with payloads test case.

**Figure 7:**
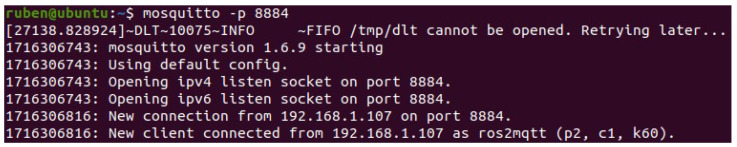
MQTT broker showing a new connection.

In this test, we explored the potential of using a robotic arm as the payload and MQTT as the communication protocol. The first step was for the user to start up the MQTT broker, which was running on an Ubuntu 20.04 virtual machine. The user then proceeded to establish a connection to the robot’s Raspberry Pi device via SSH. The next step was to initiate the UI script, as illustrated in Figure 6, and select the arm payload and the MQTT communication technology. The script kindly requested the messaging broker’s IP and port, as well as a name for the required MQTT topic. Once this process was complete, the launch file was initiated, which enabled everything to be put into operation. It was then possible to check in the messaging broker’s logs that a new client had connected (Figure 7).

**Figure 8:**
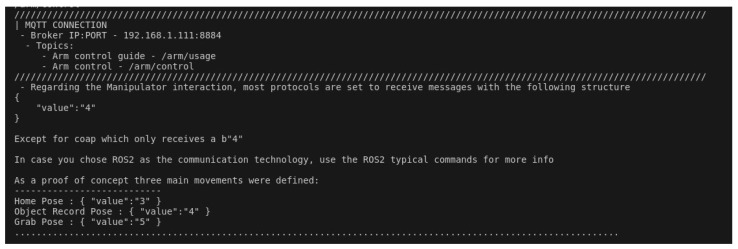
Output of the connection guide regarding the user’s choices.

**Figure 9:**
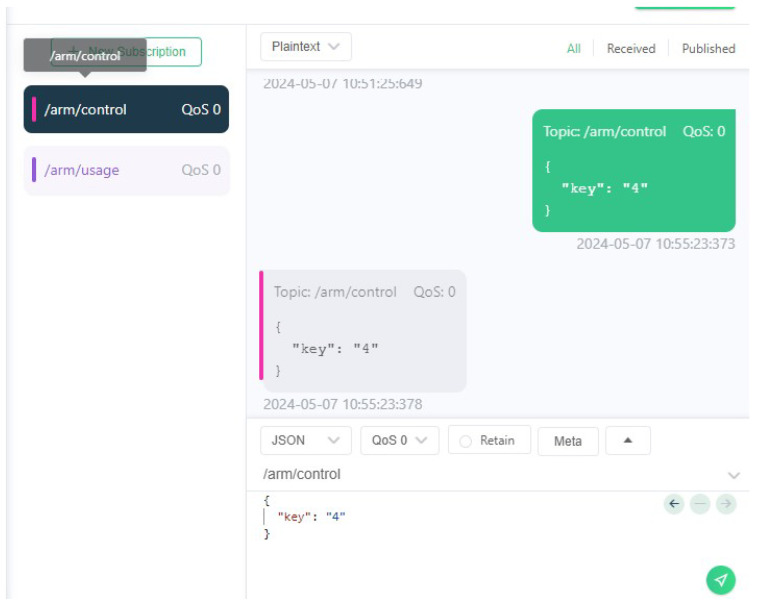
Using MQTTX to publish arm control messages.

**Figure 10:**
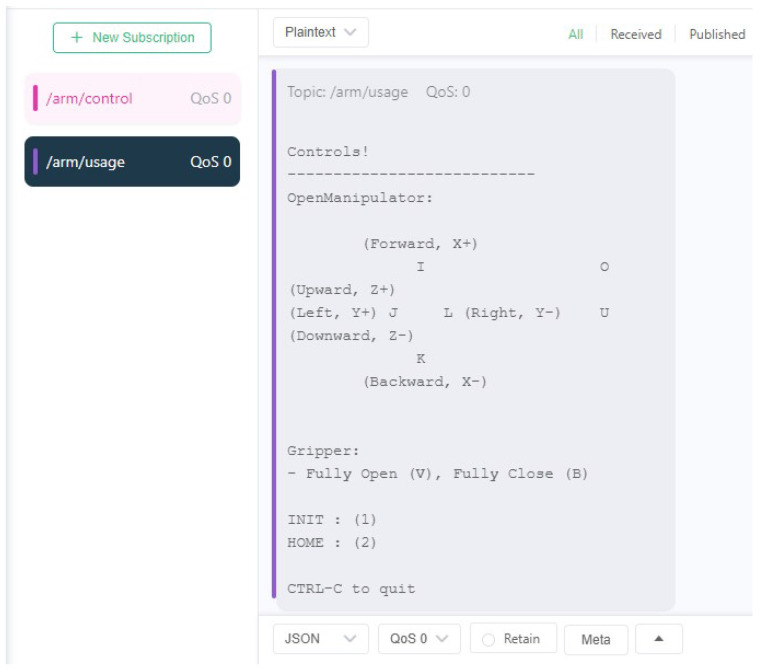
Using MQTTX to subscribe to the arm guide topic.

A software called MQTTX, which is a simple MQTT client running on the user’s local machine, was used to establish a connection to the same messaging broker. Then, with the assistance of the UI script (Figure 8), the user was able to initiate the publication of control messages (see Figure 9) to the newly created MQTT topic, which resulted in the desired movement of the robotic arm. For the purposes of this test case, the ROS2 arm payload package also published control guidelines through an MQTT topic, as can be seen in Figure 10.

### 4.2. The Arm Manipulation through Multiple Communication Technologies Test Case

**Figure 11:**
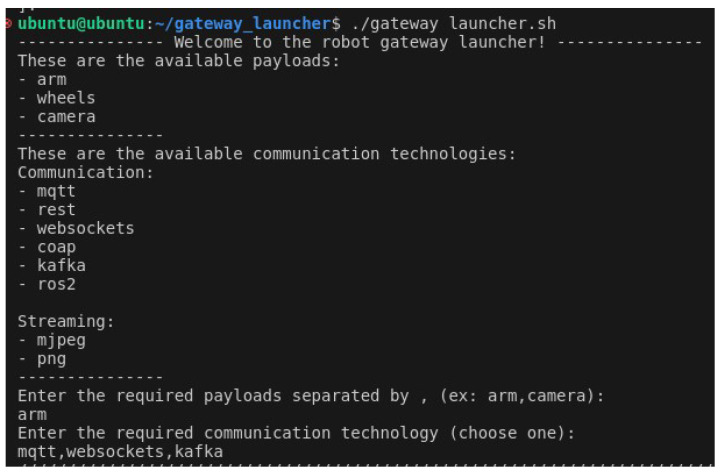
UI showing the payloads and technologies selection process.

**Figure 12:**
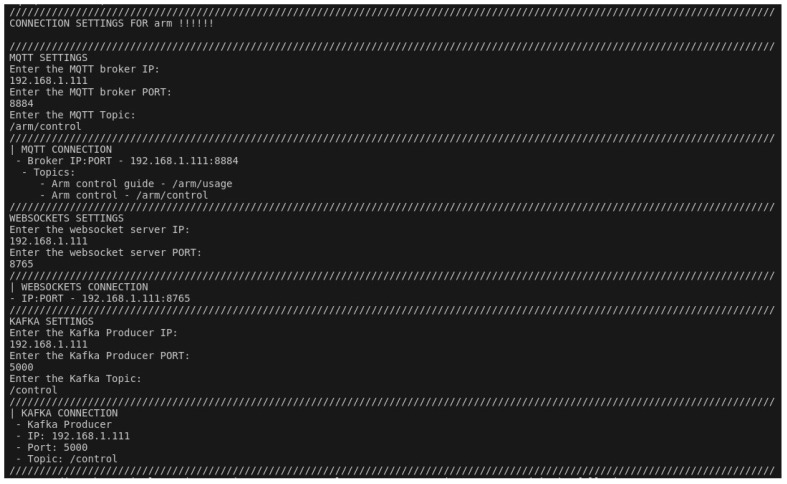
UI showing the connection settings provided by the user.

**Figure 13:**
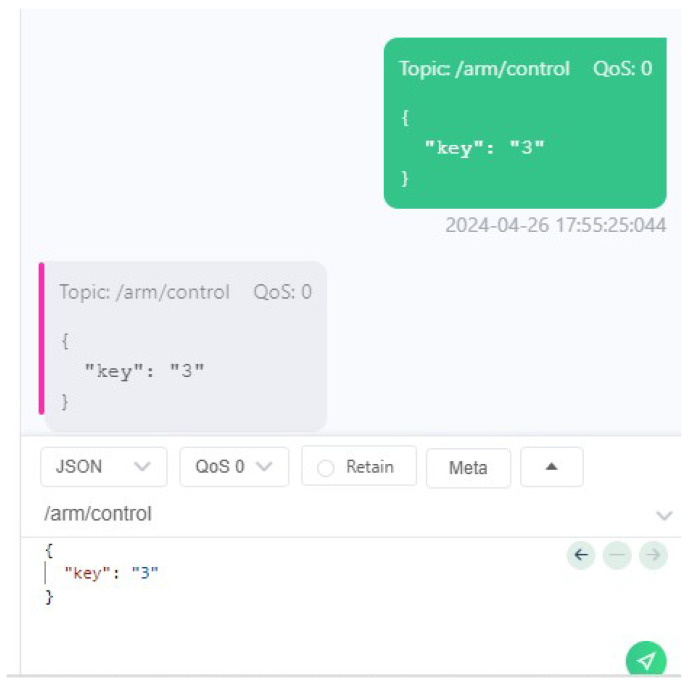
Usage of MQTTX to test the MQTT communication.

**Figure 14:**
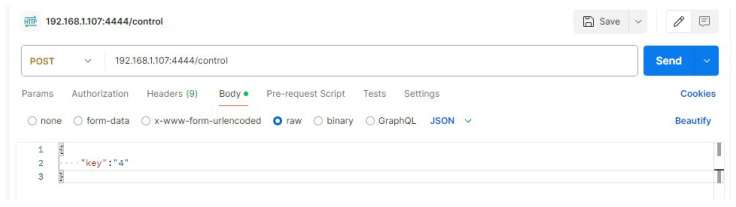
Usage of Postman to test the Websockets communication.

**Figure 15:**
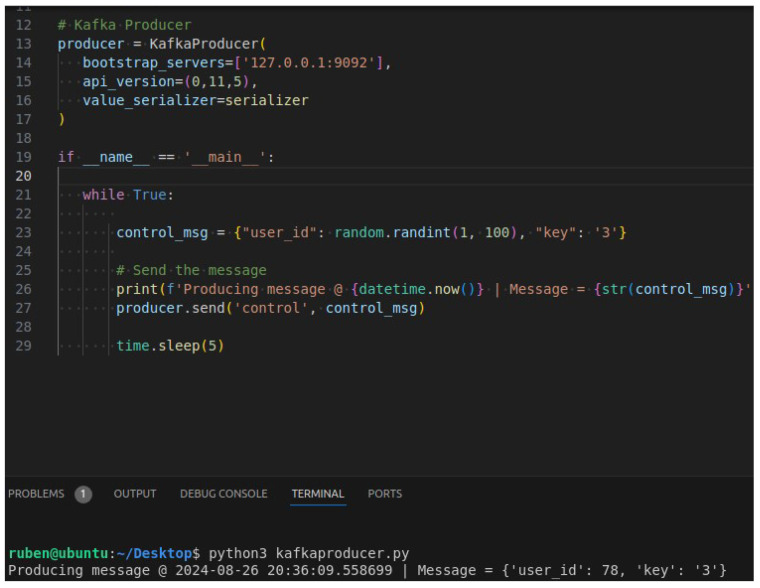
Usage of a python script to test the Kafka communication.

**Purpose:** The aim of this test case is to ascertain whether the user can launch the UI script and select the arm payload and multiple high-level communication protocols in a seamless manner, and whether the arm can be controlled by all the selected communication technologies simultaneously.
**Test Bed:** Table 2 refers to the resources used for this test case.
**Results:** In this test case, the user has chosen MQTT, websockets and Kafka as can be observed in Figure 11 and Figure 12. After having everything running, the MQTTX (Figure 13) was used to communicate through MQTT, Postman was used for websockets (Figure 14), a python script was employed for Kafka communication (Figure 15) and everything has worked as expected.

Hence, the arm moved each time a new message was received, regardless of the technology used to send control messages. This test offers an opportunity to assess the flexibility this solution provides in terms of communication.

### 4.3. The Simulation of an Object Grab Test Case

**Figure 16:**
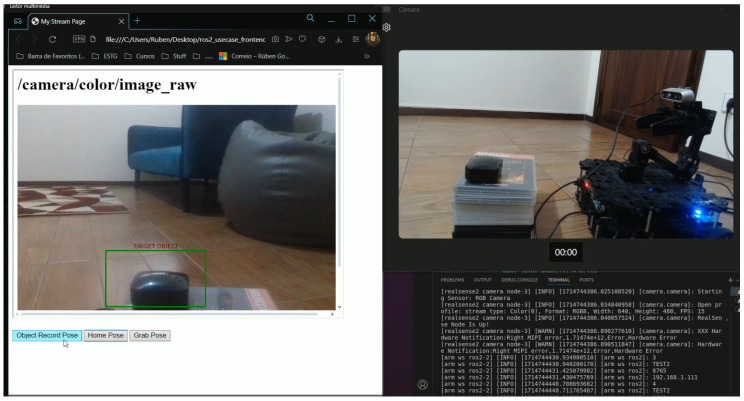
Web page receiving the camera stream and controlling the robotic arm through websockets.

**Figure 17:**
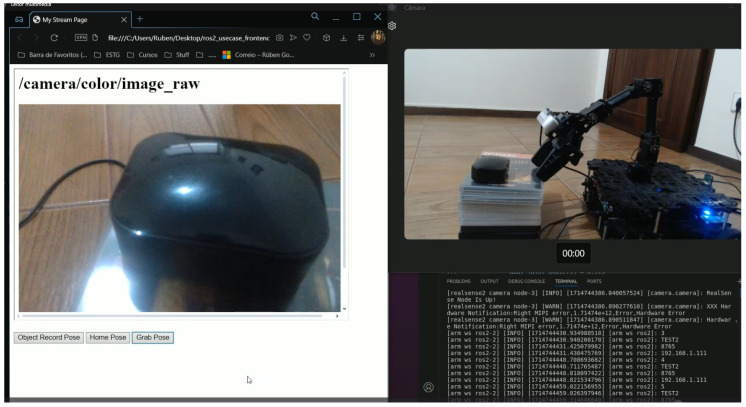
The robot arm reaching the target object controlled from the client web page.

**Purpose:** This test case aims to observe and touch an object. To this end, a web interface was created that allows the user to visualise the images acquired by a real sense camera attached to the robot arm as well as to manipulate the arm in order to reach the object. In this use case, websockets were used for the interaction between the user and the robot.
**Test Bed:** Table 3 contains all the resources used for the execution of this test.
**Results:** Once the websockets server was activated, the arm and camera payloads and websockets were selected at the gateway’s initial configuration, as part of this preliminary test. Subsequently, all the connection settings were provided and everything proceeded without incident. Once all the necessary packages had been launched, the UI script was able to print the connection data for each of the launched payloads. At this point, an HTML web client was created. In this web client, an iframe object was created and its source attribute was set to the stream’s URL. Subsequently, some HTML buttons were created for each of the arm’s defined poses, with each button sending a different message through WebSockets. This resulted in a web page (Figure 16) receiving the camera stream where the target object was visible and in three buttons that, once clicked, would move the arm until it reached the object (Figure 17).

Once everything was up and running, it was possible to observe the object through the camera and see the captured frames from the web interface as well as move the arm towards the target object through the HTML buttons.

### 4.4. The SmartFarm Project Gateway Test Case

**Figure 18:**
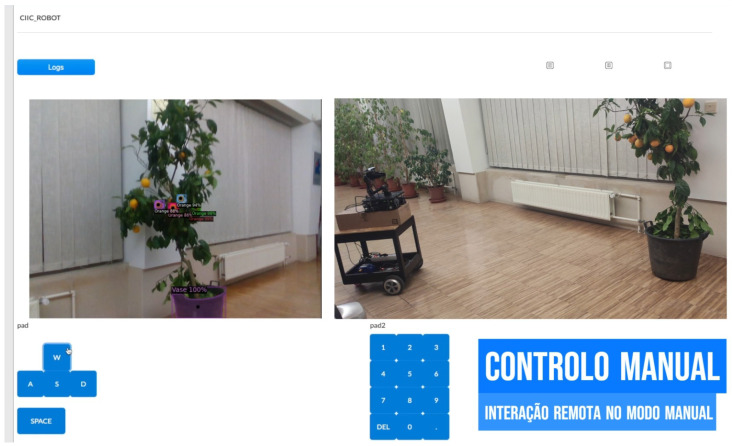
ROS2-based The robot being remotely controlled through websockets.

**Figure 19:**
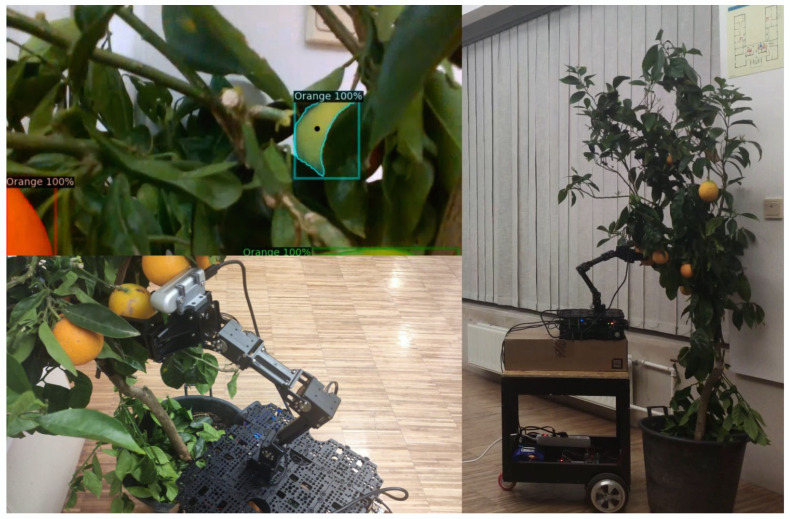
The camera detecting oranges and the arm moving onto each one.

**Purpose:** This test brings together the work of different researchers in the same research group to create a controlled multi-component real-world scenario. The objective is to integrate cloud robotics and computer vision research with artificial intelligence through the ROS2-based gateway to enable a robot prototype to move towards an orange tree and grab oranges with a robotic arm. The user must first connect to the robot via the cloud robotics platform and activate the wheels, camera and arm payloads. Activate the payloads and remotely control the wheels from the cloud robotics platform with the aid of the gateway. Once the robot reaches the tree, the camera payload observes the orange tree and sends the captured frames to an external websockets server via the ROS2-based gateway. The server then uses the computer vision AI model to detect all the oranges and extract their relative coordinates, which are sent back via websockets to the ROS2-based gateway. The gateway then converts the coordinates messages into the ROS2 message structure supported by the arm so that the arm can move towards each orange.
**Test Bed:** Table 4 enumerates all the resources required when conducting this test.
**Results:** In this test case, the gateway was responsible for interacting with the robotic arm, the robot’s wheels, camera and to send and receive websockets messages while being integrated into a more complex test case, where a cloud robotics platform was used as well as an AI model to recognise oranges in an orange tree. The robot was manually guided to approach the small orange tree; then, the orange picking/touching operation was totally automated and realised with success (Figure 18 and Figure 19). Employing an AI model to detect orange trees would also replace the manual approaching operation by a totally automated task of visiting orange trees in an orchard and picking/touching the oranges.

## 5. Conclusions

This project was designed and implemented with the clear goal of contributing to several key aspects of robotics. These include robot interoperability, on-demand task adaptation, hardware modularity and resource optimisation. This project was driven by the need to challenge the current paradigm, which requires entire systems/environments to adapt to a given robot. We believe it would be more practical if things were the other way around. The gateway proposed in this article offers users and programmers the flexibility to specify which payloads they want to activate (modular hardware) depending on the task at hand and the high-level protocols they wish to use to interact with the activated payloads (interoperability). This approach allows for the optimisation of hardware resources (only the necessary payloads are activated), as well as enabling the programmer/user to utilise high-level communication protocols (such as RESTful, Websockets, Kafka, etc.), rather than low-level programming, to interact with the activated payloads.

A thorough analysis of the current literature and state of the art reveals a glaring absence of relevant information. The only comparable aspect found in other articles relates to hardware modularity on robots from a different approach: the physical form adapts to the robot’s mission rather than managing its on-demand required payloads. It is clear that the challenges mentioned here are largely overlooked, making this solution a highly promising representation of possible improvements in the field of robotics.

The architecture of this solution defines a clear division of areas within the ROS2 domain of the gateway. This division is essential for defining the purpose of each component and promoting reusability.

We have identified several next steps for future enhancements to the gateway. These include adding more high-level communication protocols to increase the level of interoperability, forming a more automated way of adding ROS2 packages for both payloads and communication protocols. This will facilitate the scaling of the gateway and promote better code reusability. We also plan to make further improvements to the UI component to detect and adapt to changes in the gateway architecture.

In addition, we are looking into ways to streamline the provisioning of payloads and communication technologies. Having to choose from multiple options can be overwhelming, so we want to make this process more efficient.

## Figures and Tables

**Table 1:** Gateway’s first test resources.

Hardware	Raspberry Pi 4 Compute Module
	Turtlebot Waffle
	Open Manipulator X
	OpenCR Microcontroller
	Windows 10 Machine
Software	Linux Ubuntu 20.04
	ROS2 Foxy
	MQTT Broker
	MQTTX
	Robotis OpenManipulator ROS2 package [20]
	Visual Studio Code

**Table 2:** Gateway multi-communication test resources.

Hardware	Raspberry Pi 4 Compute Module
	Turtlebot Waffle
	Open Manipulator X
	OpenCR Microcontroller
	Windows 10 Machine
Software	Linux Ubuntu 20.04
	ROS2 Foxy
	MQTT Broker
	MQTTX
	Postman
	Python Script
	Robotis OpenManipulator ROS2 package [20]
	Visual Studio Code

**Table 3:** Hypothetical gateway use case test resources.

Hardware	Raspberry Pi 4 Compute Module
	Turtlebot Waffle
	Open Manipulator X
	Camera Intel Realsense d435i
	OpenCR Microcontroller
	Windows 10 Machine
Software	Linux Ubuntu 20.04
	ROS2 Foxy
	HTML Web page
	Webserver ROS2 package [19]
	Robotis OpenManipulator ROS2 package [20]
	IntelRealSense ROS2 package [21]
	Visual Studio Code

**Table 4:** Second test resources.

Hardware	Raspberry Pi 4 Compute Module
	Turtlebot Waffle
	Open Manipulator X
	Camera Intel Realsense d435i
	OpenCR Microcontroller
	ESP32 Microcontroller
	Developed Wheels prototype
	Fedora Linux Machine
	2x Windows 10 Machine
Software	Linux Ubuntu 20.04
	ROS2 Foxy
	Cloud Robotics Platform
	Artificial Intelligence Model
	Robotis OpenManipulator ROS2 package [20]
	IntelRealSense ROS2 package [21]
	Visual Studio Code

## Data Availability

Data is contained within the article.
